# A novel immunochromatographic strips assay for rapid and simple detection of systemic lupus erythematosus

**DOI:** 10.1038/s41598-020-71137-0

**Published:** 2020-08-25

**Authors:** Yuhan Sun, Zhi Li, Wei Liang, Yanlong Zhang, Wanli Song, Jiazhe Song, Kai Xue, Meiling Wang, Wenying Sun, Jianguo Gu, Ming Li, Wenzhe Li

**Affiliations:** 1 College of Basic Medical Science, Dalian Medical University, 9-Western Section, Lvshun South Road, Dalian, 116044 Liaoning China; 2 Clinical Laboratory, Dalian Municipal Central Hospital, 826-Xinan Road, Shahekou District, Dalian, 116033 Liaoning China; 3 Department of Wildlife Medicine, College of Wildlife Resources, Northeast Forestry University, 26-Hexing Road, Harbin, 150040 Heilongjiang China; 4 Institute of Molecular Biomembrane and Glycobiology, Tohoku Medical and Pharmaceutical University, Sendai, Miyagi 981-8558 Japan

**Keywords:** Biochemistry, Biomarkers

## Abstract

Systemic lupus erythematosus (SLE) is a complex multi-system autoimmune disease. Detection of anti-nuclear antibodies (ANA) is fundamental for the diagnosis of SLE. In the present study, we found that the level of core fucosylation catalyzed by α1,6-fucosyltransferase (Fut8) is markedly up-regulated on immunoglobulin G (IgG) in the sera of SLE patients detected by *Aspergillus oryzae* lectin (AOL) blot. In sandwich Dot enzyme-linked immunosorbent assay (Dot-ELISA), the core fucosylation level was also found significantly increased in the sera from SLE patients with a higher ANA titer. To establish a rapid and sensitive laboratory test for the diagnosis of SLE, we prokaryotically expressed AOL and C3-D1-C3-D2-C3 of protein G (SpG3), and generate AOL-conjugated colloid gold immunochromatographic strips (ICS). The detection limit of core fucosylated IgG was 10 μg/mL for AOL-conjugated colloid gold ICS. As well as indirect immunofluorescence, the AOL-conjugated colloid gold ICS showed reliable results in the serum of 39 SLE patients. Our results indicated that the AOL-conjugated colloid gold ICS could serve as a rapid test for the detection of ANA and suspected cases of SLE.

## Introduction

Systemic lupus erythematosus (SLE) is a disease of multifactorial genesis, which is characterized by clinical manifestations of SLE, the production of autoantibodies and the formation of immune complexes^1^. The SLE pathogenesis is associated with abnormal apoptosis and accumulated nuclear material of apoptotic cells leads to antinuclear antibodies (ANAs) production and formation of circulating and/or tissue-bound IgG immune complexes^2^. Indirect immunofluorescence (IIFA) and enzyme immunoassay are widely used methods to detect the ANAs^3^. To date, methods involved immunochemical probe^4^, mass spectrometry^5^ and flow cytometry test^6^ were utilized to detect SLE. Moreover, lectin-ELISA using the fucose-lectin, Aleuria aurantia lectin (AAL), could detect the fucosylated *N*-glycan^7^. However, these tests need more complex and expensive requirements that limit its application to SLE patients. It is critically necessary to establish a rapid and sensitive laboratory test for the diagnosis of SLE.

**Figure 1:**
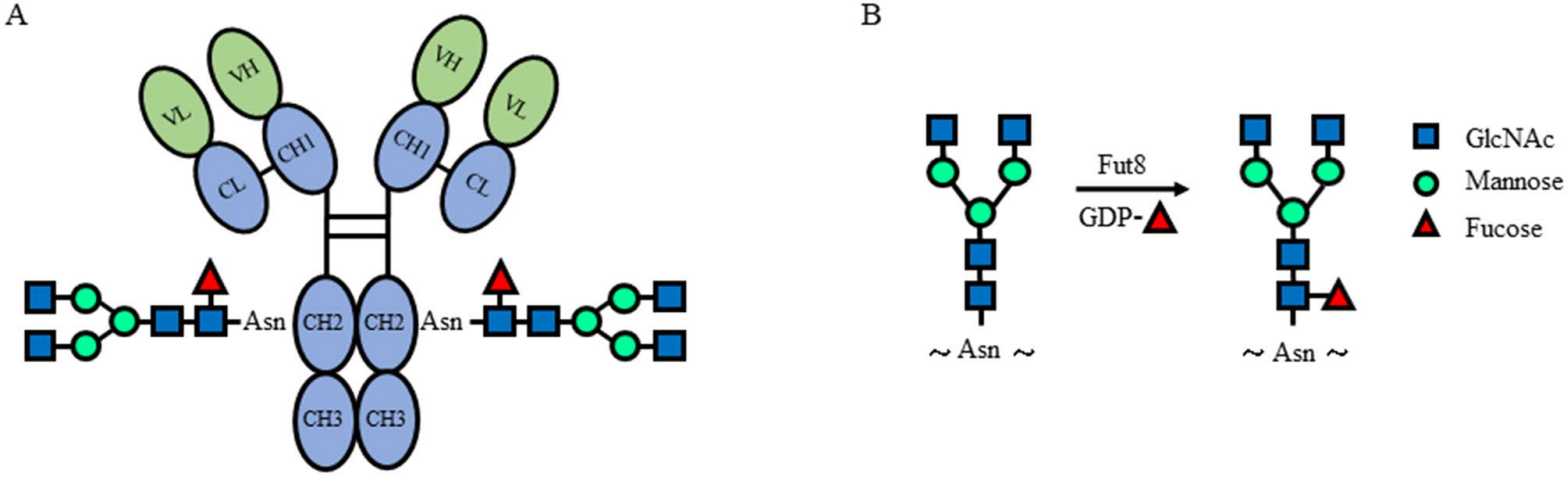
Core fucosylated N-glycan on a human IgG molecule. (**A**) The N-linked glycan at position N^297^ attached to each of the heavy chains is shown. N-glycan consists of fucose, GlcNAc, galactose and mannose residues. (**B**) Synthetic pathway for the core fucosylation catalyzed by Fut8.

Immunoglobulin G (IgG) is one of the most abundant immunoglobulins (about 10–20% of plasma protein) in human serum. IgG is composed of two heavy (H) chains and two light (L) chains. Each heavy chain consists of a N-terminal variable domain (VH) and three constant domains (CH1, CH2, CH3); the light chains consist of a N-terminal variable domain (VL) and a constant domain (CL). Indeed, the IgG is a glycoprotein. A highly conserved N-linked glycan at asparagine 297 (Asn^297^, N^297^) is covalently linked at the interface between the two CH_2_/CH_3_ forming the “fragment crystallizable’’ (Fc) of an IgG molecule (Fig. 1A). The carbohydrate moiety exists in about 5% of the total serum IgGs, and over 30 different covalently attached glycans have been detected at this glycosylation site^8^. The N-linked glycans of human glycoprotein are complex glycans, including N-acetylglucosamine (GlcNAc), mannose, galactose, sialic acid and fucose^9^. Structural evidence suggests that the glycan at N^297^ of IgG is important to maintain the conformation of Fc fragment and plays a critical role in the biological function of IgGs^10^, and thereby may critically contribute to development of autoimmune pathology. For instance, sialylation of IgGs attenuates the development of autoimmune disease^11^. De-galactosylated IgG autoantibodies are associated with severity of autoimmune disorders, such as rheumatoid arthritis (RA) and SLE^9,12^. In multiple sclerosis, polymorphisms in the gene coding for the glycosylation enzyme MGAT5 were correlated to disease severity^13^. In IgA nephropathy (IgAN), galactose-deficient IgA1 is targeted by anti-IgA1 auto-antibodies and form autoimmune complexes which induce renal toxicity by complement activation^14^.

Indeed, IgG is a heavy core fucosylated protein. In a MALDI-TOF-MS analysis, the most abundant glycans on IgG were complex types, i.e. FA2G1 (31%), FA2G2 (23%), FA2G2S1 (13%), FA2 (10%) and FA2BG1 (5%)^15^. Overall, 92% of the total IgG pool was core fucosylated, 13% bisected, 18% monosialylated and 3% disialylated^7^. α1,6-Fucosyltransferase (Fut8) catalyzes the transfer of a fucose from GDP-fucose to innermost N-acetylglucosamine (GlcNAc) of N-glycans via an α1,6-linkage to form core fucosylation^16^ (Fig. 1B). Core fucosylation plays important roles in many biological processes^17^ and altered fucosylation of glycoproteins is associated with serious diseases including cancer, cystic fibrosis, and inflammation. In the previous study, we showed that hyper core fucosylation is closely associated with the ANA production^18^. Therefore, we considered that hyper core fucosylation of IgG may contribute to SLE pathogenesis.

Immunochromatographic strips (ICS) is a rapid, convenient detection of analytes and easily visual endpoint. It is wildly used in medical fields for disease screening^19^. In the present study, we established a rapid and sensitive ICS using prokaryotically expressed *Aspergillus oryzae* lectin (AOL) and C3 domain repeated protein G (SpG3), which is specific for L-fucose recognition of serum IgG^20^ for the diagnosis of SLE.

## Results

### Core fucosylation is significantly upregulated on the IgG of SLE patients

**Figure 2:**
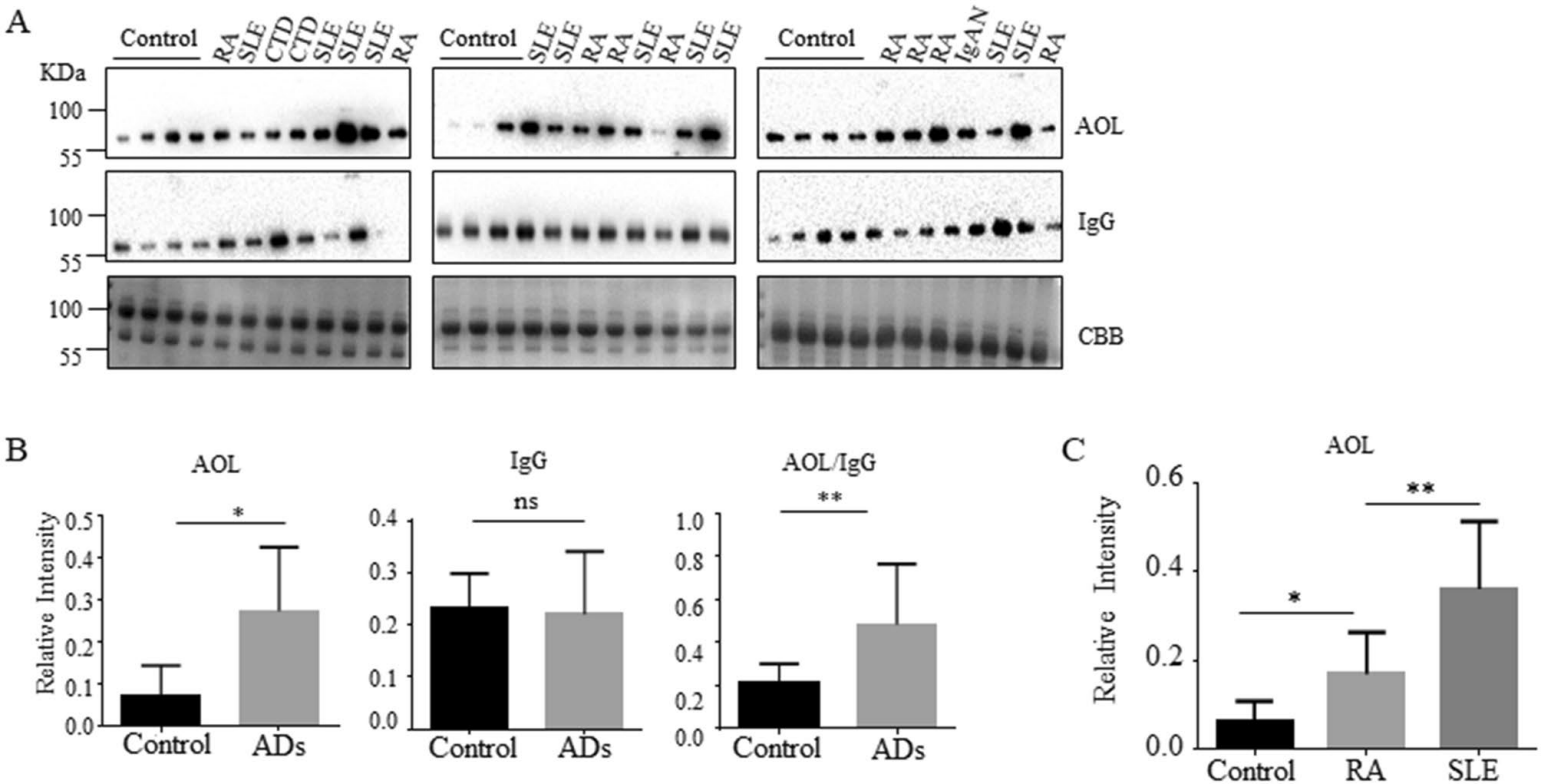
Core fucosylation was significantly increased in the AD patients. (A) Sera of AD patients were analyzed by AOL blot. Plates were incubated with biotin-conjugated AOL (1:15,000) or donkey anti human IgG (1 : 5,000), and staining with coomassie brilliant blue (CBB). Data were obtained in three independent experiments. (**B**) Densitometric analysis of the bands of IgG and AOL in AD sera. The core fucosylation levels between AD serum and health control serum were compared. The value of AOL/IgG in AD patients was analyzed. Data are shown as mean values ± SEM (*p < 0.05; **p < 0.01). (**C**) AOL blots with SLE sera and RA sera. Data are shown as mean values ± SEM (*p < 0.05; **p < 0.01).

The fucosylation modification was significantly increased in sera from AD patients^6,21^. We also detected the level of core fucosylation in the sera of AD patients and found that core fucosylation was significantly increased in AD patients, evidenced by lectin blot with AOL, which is specific for L-fucose recognition (*p < 0.05) (Fig. 2A,B). The value of AOL/IgG was markedly increased in AD patients (**p < 0.01) (Fig. 2B). The core fucosylation on the IgGs from SLE patients was higher than those from RA patients (**p < 0.01), albeit the core fucosylation was significantly increased in the serum of RA and SLE patients compared with the healthy controls (Fig. 2C).

**Figure 3:**
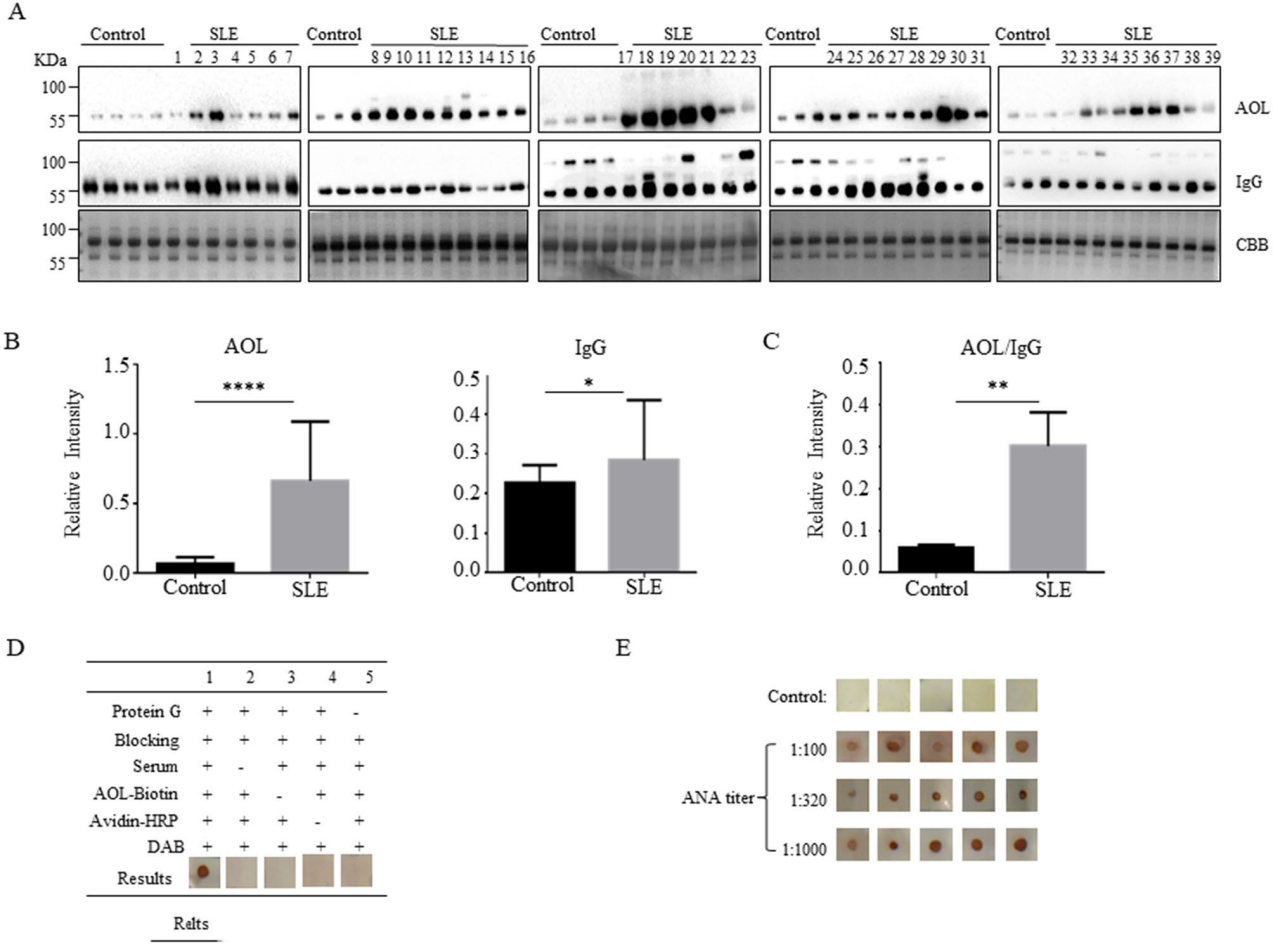
Core fucosylation was significantly increased in the SLE patients. (**A**) The sera of SLE patients were analyzed by AOL blot and Western blot. Plates were incubated with biotin-conjugated AOL (1:15,000) or donkey anti human IgG (1:5,000), and CBB. Data were obtained in three independent experiments. (**B**) Densitometric analysis of the bands of IgG and AOL in SLE sera. The core fucosylation levels between SLE serum and health control serum were compared. Data are shown as mean values ± SEM (****p < 0.0001; ***p < 0.001). (**C**) The value of AOL/IgG in SLE. Data are shown as mean values ± SEM (**p < 0.01). (D) Dot-ELISA assay. 2 μg protein G was spotted onto each membrane, and the membranes were incubated successively with serum (1:100), biotin-conjugated AOL (1:1,000) and HRP-conjugated streptavidin (1:3,000). 1, positive control serum; 2, negative control without serum only; 3, negative control without biotin-conjugated AOL only; 4, negative control without HRP-conjugated streptavidin only; 5, negative control without protein G immobilized. (**E**) SLE sera were detected by Dot-ELISA assay. SLE sera were separated with different ANA titers, 1:100, 1:320, 1:1,000 (n = 5). Each dot represents one single of patients who were previously tested with different titers of ANA.

To further investigate the level of core fucosylated IgG in the SLE sera, we detected the sera of the SLE patients (n = 39) by AOL blot. The level of core fucosylation was significantly increased in SLE serum (****p < 0.0001), and the expression of IgGs was also upregulated (*p < 0.05) (Fig. 3A,B). The value of AOL/IgG was dramatically increased in SLE patients (**p < 0.01) (Fig. 3C). Moreover, IgGs from the serum of SLE patients and healthy controls were purified with Protein A Magnetic Beads. Indeed, the core fucosylated N-glycans were increased on the IgGs of the SLE patients compared with those of healthy controls by electrospray ionization mass spectrometry (ESI–MS) (Supplementary Fig. 1). These results implied that the hyper core fucosylation on the IgG is characterized in the serum of SLE patients.

Next, the Dot-ELISA was developed for the detection of core fucosylated IgG in the SLE sera. Dot-ELISA showed that 2 μg/dot Protein G concentration, 1:100 dilution of serum, 1:1,000 dilution of biotin-conjugated AOL and 1:3,000 dilution of HRP-conjugated streptavidin were optimized (Fig. 3D). Among the SLE clinical samples, we grouped the samples according to the ANA titer, and performed Dot-ELISA. Rates of detection of core fucosylated IgG by the Dot-ELISA were 100%, while those of healthy controls were indicated as negative result without any false-positive results (Fig. 3E).

### Construction of pET28a-FleA and expression of AOL

**Figure 4:**
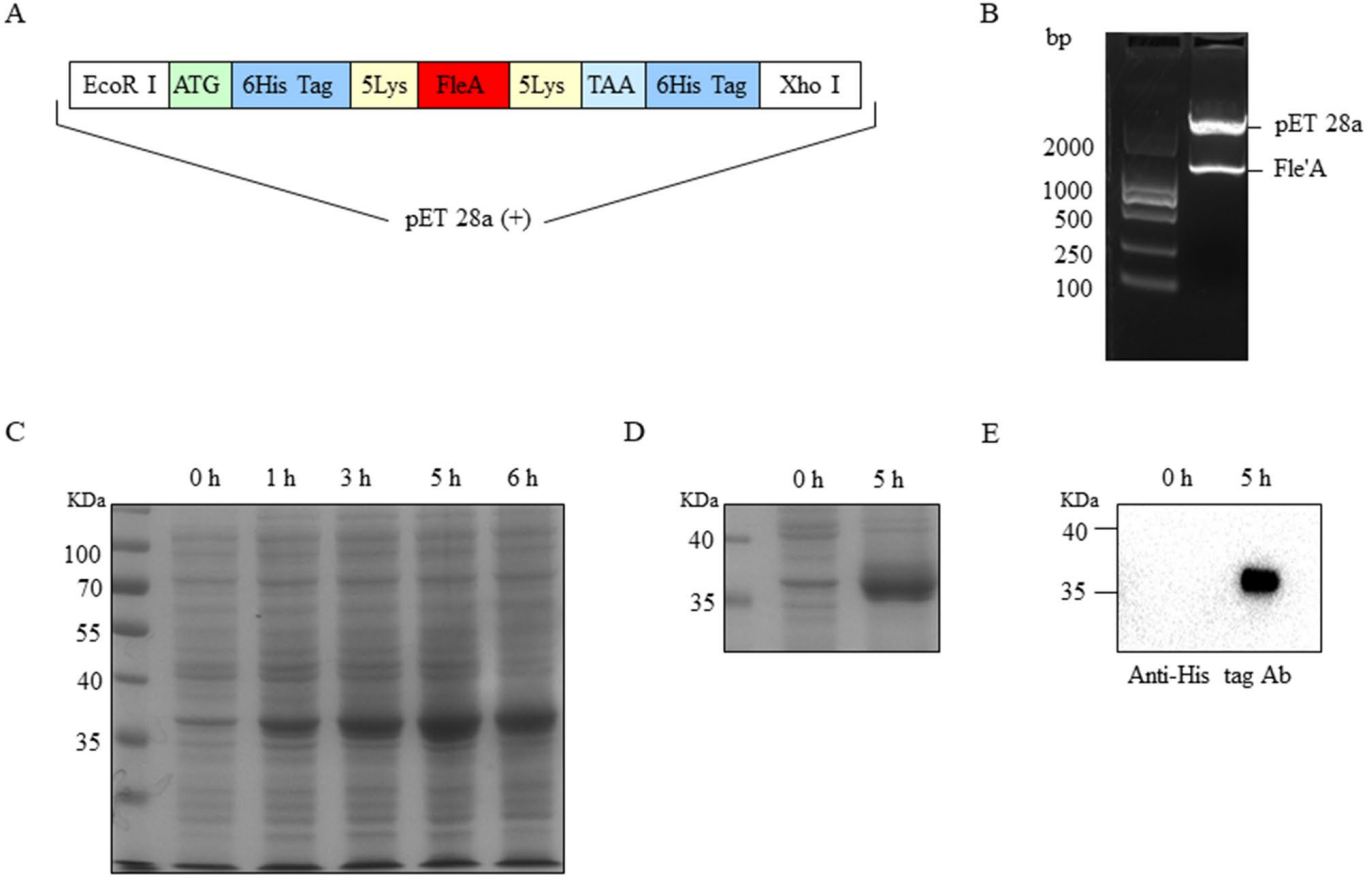
The construction of pET28a-*FleA* and the expression of AOL. (**A**) The structure of redesigned gene of *FleA*. (**B**) The detection of pET28a-*FleA* by enzyme digestion. Line 1 was marker DL2000; pET28a-*FleA* in line 2 was digested with restriction enzyme *EcoR* I and *Xho* I. (**C**) The BL21 cells were stimulated with 0.5 mM IPTG for 0, 1, 3, 5 and 6 h at 37 °C with shaking. The expressing quantity was the highest at the time of 5 h. The AOL corresponding to 36 KDa was detected by 8% SDS-PAGE. (**D**) Purification of His-tag AOL. The expressed His-tag AOL was purified by Ni-sepharose affinity chromatography. Line 1, marker; Line 2, control sample; Line 3, the purified sample with stimulation for 5 h. (**E**) Western blot. His-tag AOL was incubated with mAb against His-tag (1:3,000). Line 1, control sample; Line 2, the purified sample with stimulation for 5 h.

Given that there are six fucose-binding sites in the structure of AOL, which has the most specific binding capability to core fucose^20,22^, we prokaryotically expressed the AOL-encoding gene, *FleA*, from Aspergillus oryzae. Moreover, because the surface of colloidal gold harbors electrostatic characteristics and hydrophobic properties^23^, we added 10 lysine (lys) into pET28a-*FleA* expression system to improve the ability to conjugate with colloidal gold (Fig. 4A). Pet28a-*FleA* was confirmed by restriction enzyme (*EcoR* I and *Xho* I) digestion and a positive clone was sequenced to show the identity of *FleA* gene (Fig. 4B).

The AOL was expressed after induction of IPTG for 5 h (h) in *E. coli* BL21 (DE3) transformed with the plasmid pET-28a (+)-*FleA* (Fig. 4C). The AOL was purified by Ni^2+^-NTA affinity chromatography. SDS-PAGE results verified the successful purification of the AOL with nearly 95% purity (Fig. 4D). AOL corresponding to 36 KDa was recognized by anti His-tag Ab (Fig. 4E).

### Expression of C3-D1-C3-D2-C3 domains of protein G (SpG3)

**Figure 5:**
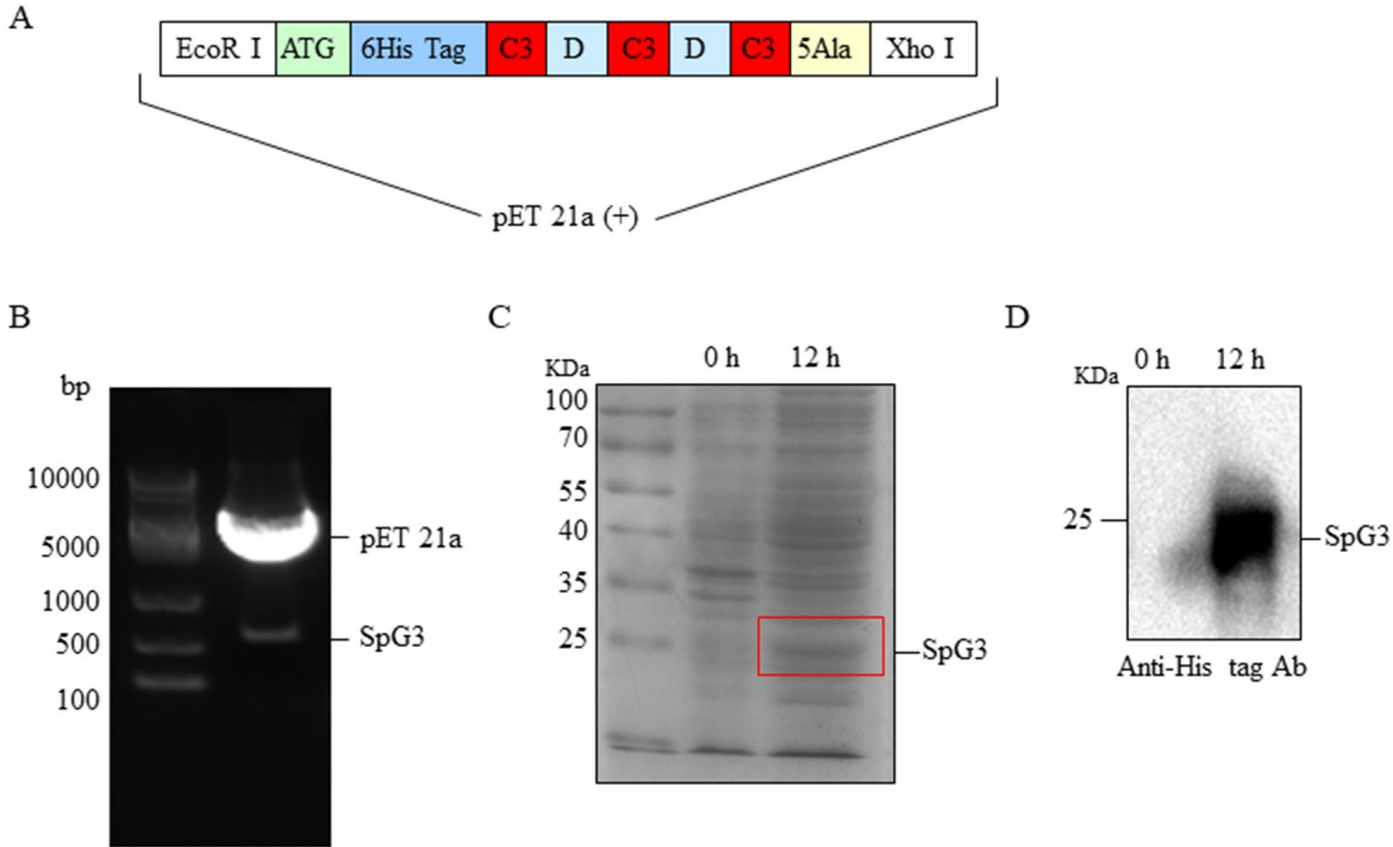
The construction of pET21a-SpG3 and the expression of SpG3. (**A**) The structure of redesigned gene of *SpG3*. (**B**) The detection of pET21a-SpG3 by enzyme digestion. Line 1 was marker; pET21a-SpG3 in line 2 was digested with restriction enzyme *EcoR* I and *Xho* I. (**C**) The expression of SpG3 induced by 0.5 mM IPTG for 12 h and shaking at 30 °C. Line 1, marker; Line 2, control sample; Line 3, the sample with stimulation for 12 h. (**D**) Western blot. The membrane was incubated with monoclonal antibodies (mAb) against His-tag (1:3,000). Line 1, control sample; Line 2, the purified sample with stimulation for 12 h.

Protein G is spontaneously released from streptococci, especially in group G streptococci, which has high affinity for binding to IgG. The C3 domain of protein G possesses a higher binding affinity to sites on the Fc portion of IgG^24^. Thus, we designed C3-D1-C3-D2-C3 domains of protein G (SpG3). Moreover, to improve the affinity for immobilizing with the NC membrane, we insert five alanine (Ala) gene after the initiation codon (ATG) and six histidine (His) gene before the terminal codon (TAA) into PU57 cloning vector (Fig. 5A). Recombined SpG3 fragments were digested with *EcoR* I and *Xho* I, and then inserted into *EcoR* I and *Xho* I-digested pET21a (+), yielding pET21-SpG3. PET21a-SpG3 was confirmed by restriction enzyme digestion (Fig. 5B). The pET21a- SpG3 vector was induced by IPTG (Fig. 5C) and a 21 KDa protein was detected by anti His-tag Ab (Fig. 5D).

### Establishment of AOL-conjugated colloidal gold Dot-ELISA

**Figure 6:**
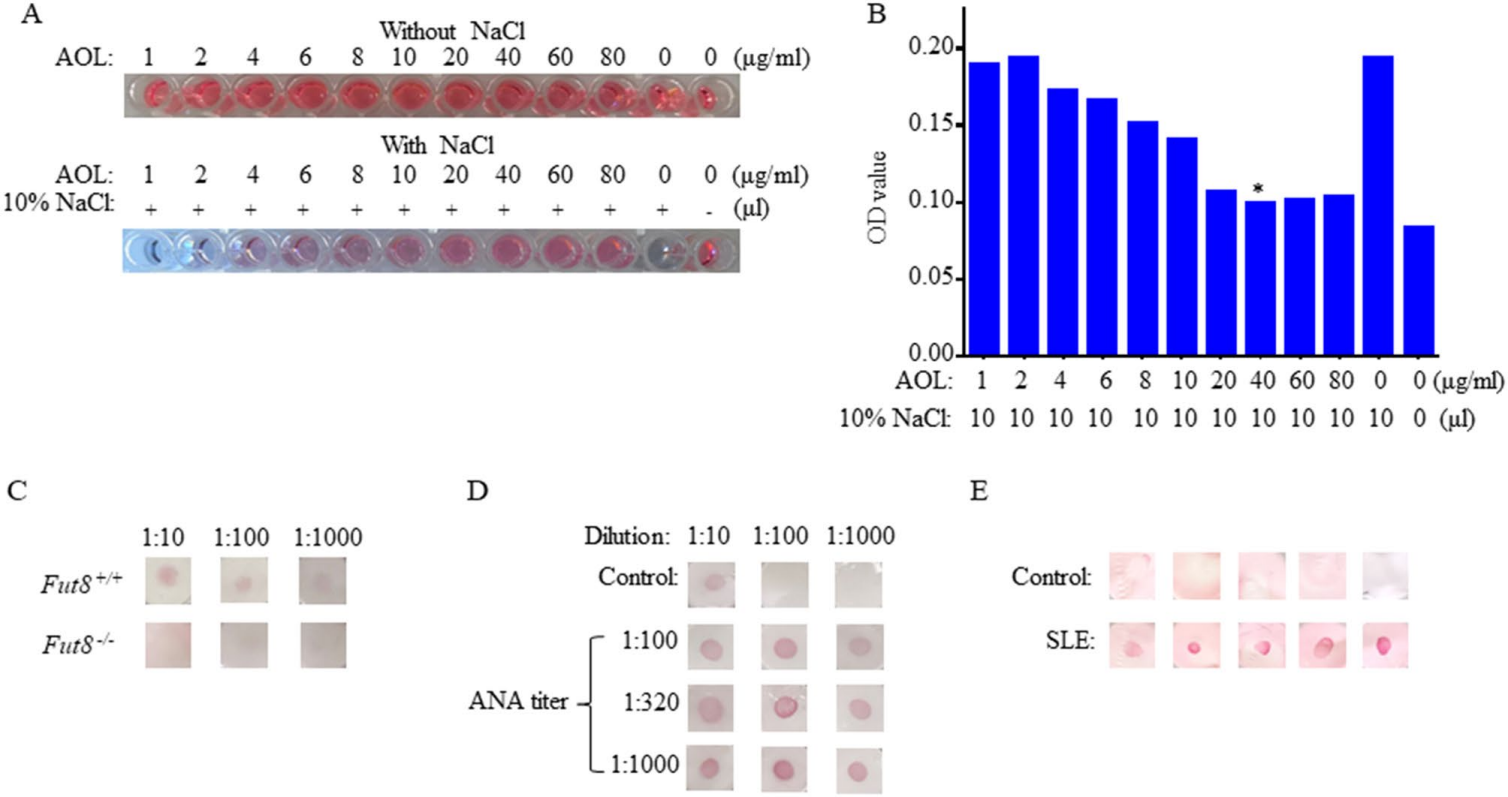
The sensitivity and specificity for detection of SLE by AOL-conjugated colloidal gold Dot-ELISA assay. (**A**) Optimal ratio of colloidal gold and AOL. To determine the optimal ratio of colloidal gold and AOL for conjugation, a fixed amount of colloidal gold (100 μL) and 10% NaCl (10 μL) was mixed with an increasing amount from 0 to 80 μg/mL, of AOL. As the concentration of the antibodies increased, the color of the AOL-conjugated colloid gold became red. (**B**) The optimization of the minimum amount of AOL for stabilizing colloidal gold solution. (**C**) The specificity of AOL-conjugated colloidal gold Dot-ELISA. The sera (dilution from 1: 50 to 1:1,000) of *Fut8*^+*/*+^ mice and *Fut8*^*−/−*^ mice were analyzed by AOL-conjugated colloidal gold Dot-ELISA. (**D**) The optimization of AOL-conjugated colloidal gold Dot-ELISA. SLE sera with different ANA titers (from 1:100 to 1:1,000) were analyzed by AOL-conjugated colloidal gold Dot-ELISA. (**E**) Detection of SLE sera by AOL conjugated colloidal gold Dot-ELISA.

Preparation of high-quality AOL-conjugated colloid gold solution is an essential step to ensure the peculiarity and sensibility of the immunological diagnosis. NaCl causes the aggregation of colloidal gold solution and changes the color from red to blue. If the color does not vary with the addition of NaCl, the AOL-immobilized on the colloidal gold solution contains the optimal concentration of AOL for fully covering the surface of all the colloidal gold solution. To obtain the AOL-conjugated colloidal gold solution, 100 μL of colloidal gold nanoparticles were mixed with 1, 2, 4, 6, 8, 10, 20, 40, 60 and 80 μg/mL AOL expressed in the addition of 10% NaCl. The optimum concentration of AOL adsorption was determined to be 40 μg/mL. At this concentration, AOL was confirmed to be the minimum amount for stabilizing colloidal gold solution (Fig. 6A,B).

FUT8 is the sole enzyme responsible for catalyzing the core fucosylation in human. As no core fucosylation can be detected in the sera of *Fut8*^*−/−*^ mice, we detected the sensitivity of this test by the different dilution of *Fut8*^+*/*+^ serum and found that the detection limit was 1:1,000 dilution in *Fut8*^+*/*+^ sera (10 μg/mL), while negative result was in *Fut8*^*−/−*^ mice sera (Fig. 6C). AOL-conjugated colloidal gold Dot-ELISA showed that 2 μg/dot SpG3 concentration, 1:100 dilution of sera, 0.2 μg of AOL-conjugated colloidal gold were optimized (Fig. 6D). Among the clinical samples from 39 SLE patients, we grouped the samples according to the ANA titer of 1:100, 1:320 and 1:1,000, and performed AOL-conjugated colloidal gold Dot-ELISA. The core fucosylation is higher in the serum of 1:1,000 ANA titer than those of 1:100 or 1:320 ANA titers, indicated the sensitivity of the assay for detection of core fucosylated IgG (Fig. 6D). We also found that the core fucosylation level was significantly increased in sera from SLE patients by AOL-conjugated colloidal gold Dot-ELISA (Fig. 6E).

### Detection of SLE by AOL-conjugated colloidal gold ICS

**Figure 7:**
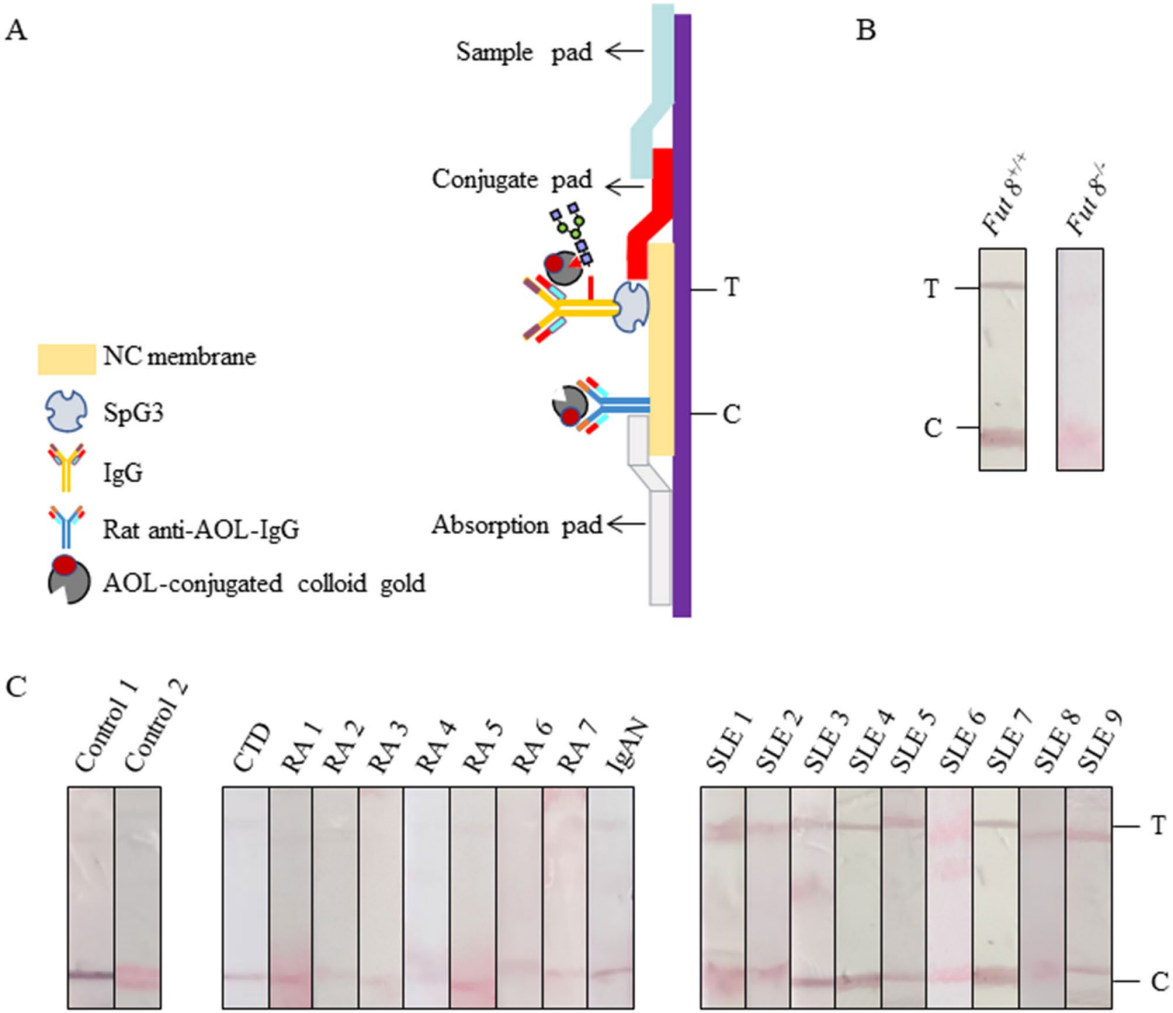
Establishment of AOL-conjugated colloidal gold ICS. (**A**) The structure of the ICS. ICS was assembled by sample application pad, AOL-conjugated colloidal gold pad, nitrocellulose membrane and the absorption pad successively. (**B**) The specificity of the AOL-conjugated colloidal gold ICS was analyzed by the serum (dilution of 1:100) of *Fut8*^+*/*+^ mice and *Fut8*^*−/−*^ mice. (**C**) Detection of SLE by AOL-conjugated colloidal gold ICS. Sera from different Ads were diluted 1:100 and detected.

We have used novel ICS concept; this is dependent on the transport of a reactant to its binding partner immobilized on the surfaces of the membrane. Capillary action of aqueous-medium through membrane pones is applied for the transfer. This helps to separate the unbound reactant from the binding complex formed at the liquid–solid interface ^25^. ICSs were assembled in sequence, which was laminated by the sample application pad, AOL-conjugated colloidal gold pad, NC membrane and the absorption pad (Fig. 7A). Samples were recorded as positive when two clear red lines appeared. And samples were considered negative when detection band was not colored but control band turned red. We firstly proved the specificity of AOL-conjugated colloidal gold ICS by the *Fut8*^+*/*+^ serum and *Fut8*^*−/−*^ serum. The serum of *Fut8*^*−/−*^ mice indicated only C line, while the serum of *Fut8*^+*/*+^ mice had both test (T) and control (C) lines (Fig. 7B). Similarly, the AOL-conjugated colloidal gold ICS for SLE sera showed positive results, but those were not in the healthy control sera (Fig. 7C). Moreover, the AOL-conjugated colloidal gold ICS showed no cross-reactivity with the other ADs, such as RA, IgAN, connective tissue disease (CTD), indicated that the AOL-conjugated colloidal gold ICS established are highly specific to SLE (Fig. 7C).

## Discussion

SLE is a sophisticated AD, and the risk of death for SLE patients is still 2 times greater than those of general population. ANAs are hallmarks of SLE. High-titer ANAs directly contribute to SLE pathogenicity by multiple mechanisms^1^. IIFA is the preferred method for SLE diagnosis in clinics. However, the method is time-consuming, very subjective and expensive^26^. Therefore, rapid and sensitive laboratory tests for the diagnosis of SLE are urgently needed to be established^27^. In the present study, we found that core fucosylation on IgG was significantly upregulated in SLE serum. The reliability and rapid method for diagnosis of SLE was successfully established by targeting the core fucosylated IgG by AOL-conjugated colloidal gold ICS.

Protein glycosylation is essential for a multitude of biological processes, as exemplified by the glycosylation of immunoglobulins. The changes of glycosylation of immunoglobulins are linked to the pathophysiology of AD pathogenesis. In addition to the differences in the number of disulfide bonds and the length and flexibility of the hinge region, changes of glycosylation profiles of IgG also are related to AD pathogenesis, such as RA and SLE^28–34^. Hypogalactosylation of IgG in the MRL/Mp-lpr/lpr lupus mice model was reported by Mizuochi et al.^35^. Interestingly, anti-inflammatory activity was observed in lupus BXSB mice after the removal of glycan moieties on N^297^ following the treatment of IgG with streptococcus endoglycosidase^36^. Similarly, another study showed that treatment by streptococcus endoglycosidase can abolish the proinflammatory properties of immune complexes from SLE patients^37^. The glycan of IgG is hyper core-fucosylated complex with bisecting N-acetylglucosamine (GlcNAc). Sjöwall^7^ found that core-fucosylated glycans was increased on the IgG of SLE patients. The hyper core fucosylation is closely associated with the ANA production^18^. In the present study, we detected core fucosylated IgG in SLE patients by Dot-ELISA with high sensitivity and specificity.

ICS is efficient and one step immunochromatographic assay. It can be carried out at the sites since it does not require use of many reagents and is performed in one step only so no need of specialized facility^38^. In the present study, we established ICS with AOL-conjugated colloidal gold to detect core fucosylated IgG of SLE patients. Preparation of high-quality colloid gold solution is an essential step to ensure the peculiarity and sensibility of the ICS test. Different sizes of gold nanoparticles have different conjugation efficiency under different concentrations of proteins^25,39^. To enhance binding affinity to colloid gold particle, we added 10 lysine (lys) to prokaryotic expressed AOL, and the appropriate amount of AOL was 40 μg/mL. In addition, the porous structure of the NC membrane provides a higher binding capacity. To improve the affinity for immobilizing with the NC membrane, we expressed C3 domain repeated SpG3 with five alanine (Ala) and six His. The use of SpG3 spotted NC membrane could greatly facilitate the reproducibility and field applicability of the ICS. The optimized dilution of clinical serum of SLE patients was 1:100 for detection of ICS generated. ICS has a lot of advantages compared to traditional assay, such as needing no equipment or trained operators and having easily readable results within 5 min. Comparison of the results of ICS and IIFA yielded a relative reliability of 100% without false-positive results in healthy controls and other ADs. The hyper core fucosylation were detected in RA patients as well as SLE in the AOL blot, but not in AOL-conjugated colloidal gold ICS. We proposed that there are two reasons why the core fucosylated IgGs in RA serum showed the negative reaction in AOL-conjugated colloidal gold ICS. First, the level of core fucosylated IgGs in RA sera is significantly lower than in SLE serum (**p < 0.01). Second, about 80–90% of RA patients have circulating rheumatoid factors, it is reasonable to suppose that the rheumatoid factors bind to the IgGs to suppress the binding of IgG to the SpG3.

In summary, a key finding of our study is that the core fucosylated IgGs could serve as a useful biomarker for SLE detection in clinical practice. In the present study, both AOL and SpG3 were prokaryotically expressed. Since the N-glycosylation pathways were lacked in bacteria, the sole glycan on the IgGs was from the sera of SLE patients. Therefore, the AOL-conjugated colloidal gold ICS showed high specificity and sensitivity. Our data pave the way for the future development of an adequate screening test in epidemiologic surveillance for suspected SLE.

## Methods

### Mice and animal immunization

*Fut8*^+*/*+^ mice and *Fut8*^*−/−*^ mice were generated by crossing heterozygous *Fut8*^+*/−*^ mice in our laboratory as previously described^18^. Mice were maintained in a room illuminated for 12 h (08:00 to 20:00) and kept at 24 °C with free access to food and water in the specific pathogen-free (SPF) laboratory animal facility of Dalian Medical University, China. Mice were subcutaneously immunized with 100 µg prokaryotically expressed AOL mixed with an equal volume of complete Freund’s adjuvant (Sigma). Two-week later, mice were immunized with 100 µg of AOL. The anti-AOL sera were collected, and IgG was purified by Protein A Magnetic Beads (Novus).

### Clinical samples

Serum samples were collected from a total of 51 patients with autoimmune diseases (ADs) including 39 SLE (34 women and 5 men with a mean age of 52 years old, ranging from 18 to 71 years old) (Supplementary Table 1), 9 RA, 1 IgAN, 2 connective tissue disease (CTD) patients (Supplementary Table 2), recruited at Dalian municipal central hospital, Dalian, China. SLE patients were validated 1982 American College of Rheumatology (ACR) as well as the 2009 Systemic Lupus International Collaborating Clinics classification criteria and investigated by using indirect immune fluorescence assay (IIFA) to detect ANA. The ANA titers of SLE patients were detected with using Anti-nuclear Antibodies IgG Kit (EUROIMMUN, Germany). The positive rate of ANA is more than 1:100. ANA fluorescence model mainly showed nuclear speckled pattern and nuclear homogeneous pattern. Serum protein concentrations were determined using a protein assay BCA kit (Pierce). Serum samples belonged to the secondary use of biological specimens of SLE patients. Serum samples from 37 sex- and age-matched normal healthy blood donors served as controls. The control serum samples also belonged to the secondary use of biological specimens of heathy donors. Because the clinical samples involved in this article belonged to the secondary use of biological specimens, the requirement of informed consent for the use of patient samples was exempted by an ethics committee (the Ethics Committee of Dalian Municipal Central Hospital).

### Antibodies

Anti His-tag antibody (Ab) (2491213) was purchased from Proteintech; biotin-conjugated AOL (A2659) was from Tokyo Chemical Industry CO, LTD; horseradish peroxidase (HRP)-conjugated donkey anti-human IgG was from Proteintech (SA00001-11); HRP-conjugated streptavidin (AB 7403) and HRP-conjugated goat anti-rabbit IgG (A0208) were purchased from Abcam.

### Western and lectin blot

Equal amounts (2 μg) of protein were run on 10% sodium dodecyl sulfate–polyacrylamide gel electrophoresis (SDS-PAGE) under reducing conditions and then transferred to polyvinylidene difluoride (PVDF) membranes (Millipore Corp.). Membranes were blocked for 2 h with 5% bovine serum albumin (BSA) in TBS-T (pH 7.5, 10 mM Tris–HCl, 0.1% Tween 20, 150 mM NaCl), and incubated with the appropriate primary Abs or lectin overnight. After washing, the blots were incubated with the corresponding secondary Abs. The proteins were visualized using an ECL system (Amersham). Density analysis was performed using Quantity One software.

### Cloning of FleA gene and expression of AOL

The total RNA was extracted from the *Aspergillus oryzae* using Trizol reagent and transcribed into cDNA with the AccuPower CycleScript RT PreMix (BIONEER). The open reading frame of the *FleA* gene was amplified by PCR using the following primers in which restriction sites were inserted as underlined below and the 5 lysine (Lys) was inserted in Italics. forward, 5′-CG GAATTC *AAA AAG AAG AAG AAG* ATG TCT ACT CCT GGC GCC C-3′ (*EcoR*I) and reverse, 5′-CG CTCGAG *CTT CTT CTT CTT CTT* CTA CTC AGC CG-3′ (*Xho*I). The PCR product was cloned into the PJET vector, PJET-FleA, was produced in *E. coli* strain DH5α and sequenced by General Biosystems Co., Ltd. (Dalian, China). After digestion with *EcoR* I and *Xho* I, the correct DNA fragment was inserted into the corresponding region of the pET28a (+) expression vector by T4 DNA ligase (Takara) to construct recombinant plasmid pET28a-*FleA*.

*Escherichia coli* BL21(DE3) (Takara) cells were used for transformation with pET28a-*FleA* plasmid, and they were grown in Luria–Bertani (LB) medium supplemented with 10 µg/mL kanamycin in shaking incubator at 37 °C until they reached to the OD of 0.6 at 600 nm^40^. After stimulated with 5 mM isopropyl β-D-1-thiogalactopyranoside (IPTG), the cells were collected by centrifugation at 7,000 rpm for 15 min at 4 °C and lysed by sonication in buffer A [pH 7.4, 20 mM sodium phosphate, 500 mM NaCl, 0.1 mM phenylmethylsulfonyl fluoride (PMSF) and 1 mM β-mercaptoethanol]. Cell lysate was centrifuged at 15,000 rpm for 15 min at 4 °C, pelleted precipitates and supernatant were analyzed by 10% SDS-PAGE separately and gels were stained with Coomassie Brilliant Blue R-250 (CBB) to visualize the expression of AOL.

### Expression of SpG3 in *E. coli* BL21 (DE3)

We designed the C3-D1-C3-D2-C3 domains of protein G (SpG3), and cloned them into PU57 by General Biosystems Co., Ltd. (Dalian, China). The target SpG3 fragment was ligated into the pET21a (+) vector by T4 DNA ligase (Takara) at the *EcoR* I and *Xho* I sites to construct recombinant plasmid pET21a-SpG3. The pET21a-SpG3 plasmid was transformed into *E. coli* BL21 (DE3) cells and induced by IPTG to express SpG3. The remainder of the protocol was the same as for the AOL expression described.

### Purification of the recombinant protein

*Escherichia coli* BL21 pellets were suspended with buffer A and subjected to sonication on ice. The total cellular proteins were then partitioned into soluble and insoluble fractions by centrifugation at 12,000 rpm for 15 min at 4 °C. The insoluble fractions obtained above were used for inclusion body isolation. After washing 3 times in buffer B (pH 8.0, 20 mM sodium phosphate, 500 mM NaCl), the pellets were solubilized in buffer C (buffer B + 250 mM imidazole). The recombinant protein was purified by Ni^2+^-NTA affinity chromatography according to the manufacture´s protocol.

### Sandwich-dot enzyme-linked immunosorbent assay (Dot-ELISA)

Dot-ELlSA was done according to a previously described method^41^ with minor modification. The nitrocellulose (NC) membrane (Millipore Corp.) was divided into squares 0.5 × 0.5 cm with a hard lead pencil. Two μg of protein G (Acro Biosystems) were dotted on separate squares and dried at 37 °C for 10 min. The strips were allowed to dry, and then the protein-binding sites were blocked with a solution of 5% BSA in PBS. A 50 μL aliquot of the clinical sera was distributed into each well and the plate incubated at 37 °C for 1 h. The plates were washed and then incubated at 37 °C for 1 h with 50 μL of the biotin-conjugated AOL and then incubated streptavidin conjugated with HRP for 1 h. A substrate solution of diaminobenzidine (DAB) in 0.1 M Tris–HCl (pH 7.4), with 0.01% H_2_O_2_ was used to color the NC membranes.

### Preparation of AOL-conjugated colloid gold

After preparing colloidal gold solution, a gold aggregation test was used to determine the optimal condition of immobilization using NaCl which caused the aggregation of the colloidal gold. 100 μL of colloidal gold solution was placed in each well of a 96 wells plate. The pH of the colloidal gold solution was adjusted with 0.1 M K_2_CO_3_ to pH 8.0 Then different quantities of AOL protein at different concentrations (0, 1, 2, 4, 6, 8, 10, 20, 40, 80 µg/mL) were added to each well. The plates were incubated under gentle mixing at room temperature (RT) for 15 min. The optimal concentration of AOL was determined by addition of 10 µL of 10% NaCl solution to each well and incubated it under gentle mixing for 15 min at RT. NaCl causes the aggregation of colloidal gold solution and changes the color from red to blue^38^.

According to the above results, appropriate amount of AOL was added drop-wise to colloidal gold solution about 10 min. The mixture was stored for 30 min at RT. After that, 10% BSA was added drop-wise to the mixture about 10 min. The mixture was stored for 30 min at RT and centrifuged at 3,500 rpm for 10 min. The supernatant was reserved and centrifuged at 12,000 rpm for 20 min. The supernatant was removed. The colloid gold labeled AOL was used for Dot-ELISA or ICS.

### Preparation of AOL-conjugated colloidal gold ICS and detection of serum samples

ICS was constructed according to the previously described method with sample application pad, AOL-conjugated colloidal gold pad, nitrocellulose membrane and the absorption pad^38^. The AOL-colloidal gold conjugates probe was added onto the glass fiber and dried at 37 °C for 2 h. SpG3 and rat anti-AOL-IgG were drawn onto the NC membrane as two discrete zones, one for control (C) and another for test (T). For each test, 100 μL of every 1: 100 diluted serum samples (1 μL serum + 99 μL PBS) was added onto the sample application pad, allowing the sample to migrate upward. After about 5 min, an image containing the color signal was showed on the strip and the results were judged by the color of the T and C lines. If both T and C lines turned red, the sample was considered as positive. If the C line turned red but the T line was no color, it was considered as negative.

### ESI–MS

MS analysis was performed using an LTQ-XL linear ion trap electrospray ionization mass spectrometer (Thermo Scientific, USA) coupled with a HPLC system, as described previously^42^. The sample loaded onto a SepPak C18 SPE column and N-glycans was collected.

### Statistical analysis

Student’s *t*-test was used for statistical analysis. Data are presented as mean values ± SEM. A probability value of p < 0.05 was considered significant. *p < 0.05, **p < 0.01, ***p < 0.001, ****p < 0.0001.

### Ethical approval and informed consent

All animal experiments were conducted in accordance with the 1996 National Institutes of Health Guide for the Care and use of Laboratory Animals and approved by the Institutional Animal Care and Use Committee of the Dalian Medical University, China. All the animal works were approved by the Ethics Committee of Dalian Medical University (approval No. AEE17013). The informed consents for the all subjects (patients as well as healthy donors) were exempted by an ethics committee (the Ethics Committee of Dalian Municipal Central Hospital) (for Acceptance No. YN2017-032-01). All experimental protocols were approved by Dalian Municipal Central Hospital Institutional Review Board, and all methods were carried out in accordance with relevant guidelines and regulations.

## Supplementary information

Supplementary information
