# Author Correction: A novel immunochromatographic strips assay for rapid and simple detection of systemic lupus erythematosus

**DOI:** 10.1038/s41598-025-18605-7

**Published:** 2025-09-05

**Authors:** Yuhan Sun, Zhi Li, Wei Liang, Yanlong Zhang, Wanli Song, Jiazhe Song, Kai Xue, Meiling Wang, Wenying Sun, Jianguo Gu, Ming Li, Wenzhe Li

**Affiliations:** 1 College of Basic Medical Science, Dalian Medical University, 9‑Western Section, Lvshun South Road, Dalian, 116044 Liaoning China; 2 Clinical Laboratory, Dalian Municipal Central Hospital, 826‑Xinan Road, Shahekou District, Dalian, 116033 Liaoning China; 3 Department of Wildlife Medicine, College of Wildlife Resources, Northeast Forestry University, 26‑Hexing Road, Harbin, 150040 Heilongjiang China; 4 Institute of Molecular Biomembrane and Glycobiology, Tohoku Medical and Pharmaceutical University, Sendai, Miyagi 981‑8558 Japan

Correction to: *Scientific Reports* 10.1038/s41598-020-71137-0, published online 25 August 2020

This Article contains errors.

As a result of errors during figure assembly the top image in Figure 3A showing SLE 24-31 was a duplication of the top left image in Figure 2A.

Additionally, in Figure 7B the image for *Fut 8*^*+/+*^ was a duplication of the Figure 7C image for SLE7, and in Figure 7C the image for SLE9 was a duplication of the image for SLE 5.

The correct Figure 3 and Figure 7 and accompanying legends appear below.

**Fig. 3:**
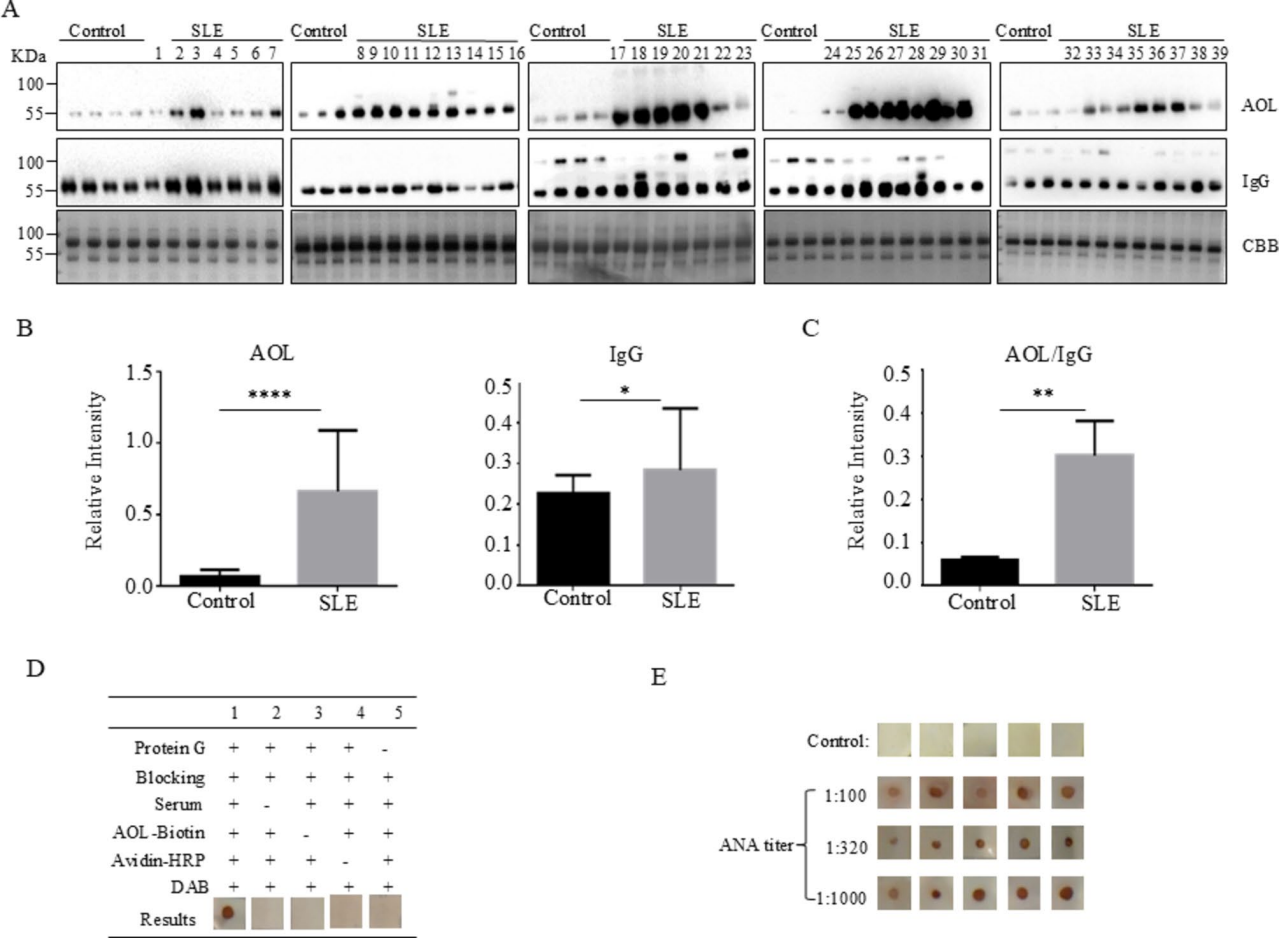
Core fucosylation was significantly increased in the SLE patients. (**A**) The sera of SLE patients were analyzed by AOL blot and Western blot. Plates were incubated with biotin-conjugated AOL (1:15,000) or donkey anti human IgG (1:5,000), and CBB. Data were obtained in three independent experiments. (**B**) Densitometric analysis of the bands of IgG and AOL in SLE sera. The core fucosylation levels between SLE serum and health control serum were compared. Data are shown as mean values ± SEM (****p < 0.0001; ***p < 0.001). (**C**) The value of AOL/IgG in SLE. Data are shown as mean values ± SEM (**p < 0.01). (**D**) Dot-ELISA assay. 2 μg protein G was spotted onto each membrane, and the membranes were incubated successively with serum (1:100), biotin-conjugated AOL (1:1,000) and HRP-conjugated streptavidin (1:3,000). 1, positive control serum; 2, negative control without serum only; 3, negative control without biotin-conjugated AOL only; 4, negative control without HRP-conjugated streptavidin only; 5, negative control without protein G immobilized. (**E**) SLE sera were detected by Dot-ELISA assay. SLE sera were separated with different ANA titers, 1:100, 1:320, 1:1,000 (n = 5). Each dot represents one single of patients who were previously tested with different titers of ANA.

**Fig. 7:**
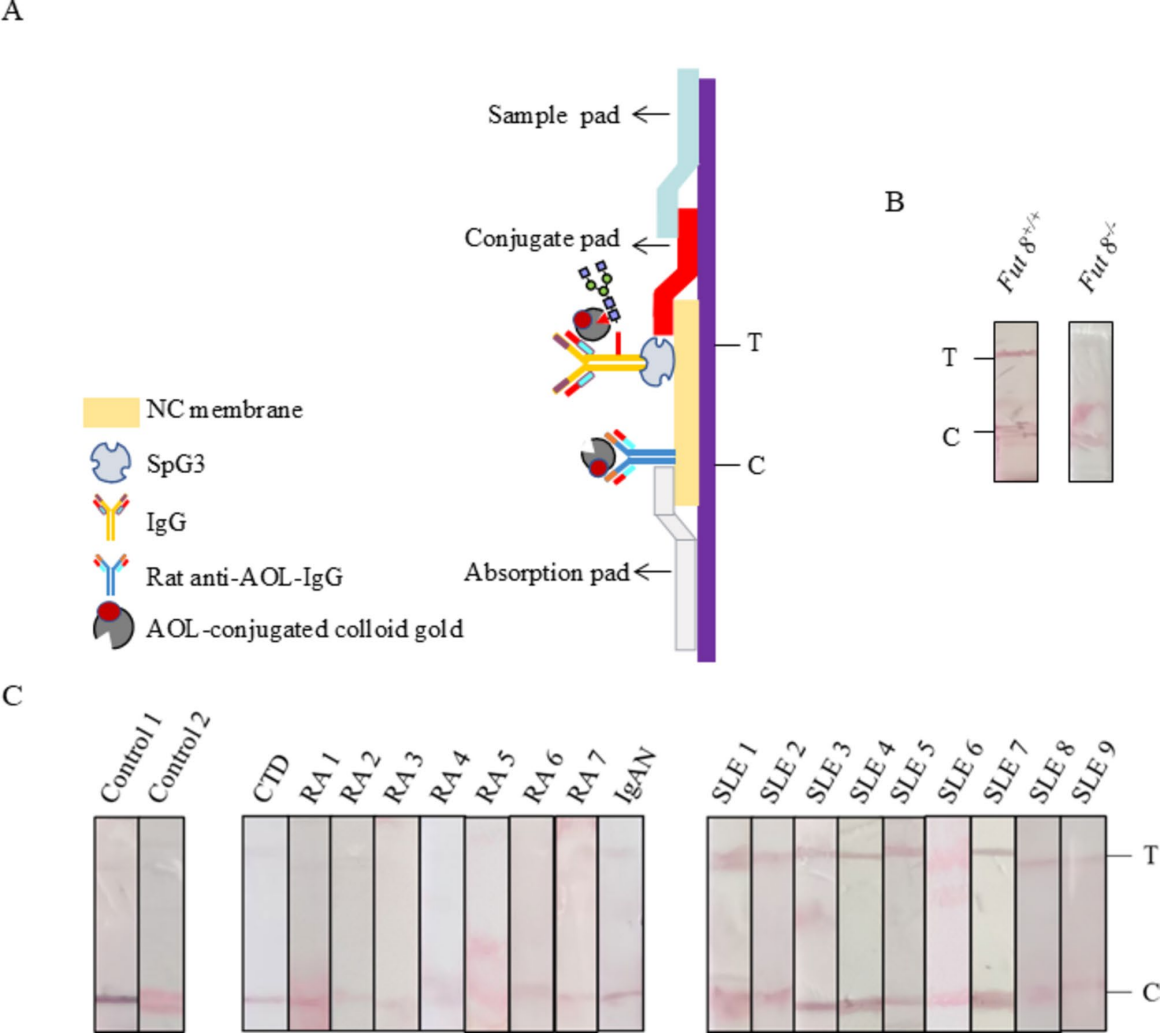
Establishment of AOL-conjugated colloidal gold ICS. (**A**) The structure of the ICS. ICS was assembled by sample application pad, AOL-conjugated colloidal gold pad, nitrocellulose membrane and the absorption pad successively. (**B**) The specificity of the AOL-conjugated colloidal gold ICS was analyzed by the serum (dilution of 1:100) of *Fut8*^+*/*+^ mice and *Fut8*^*−/−*^ mice. (**C**) Detection of SLE by AOL-conjugated colloidal gold ICS. Sera from different Ads were diluted 1:100 and detected.

