# Supplementary material for: A novel immunochromatographic strips assay for rapid and simple detection of systemic lupus erythematosus
Source: Sci Rep. 2020 Aug 25;10:14178. doi: 10.1038/s41598-020-71137-0 (PMC7447788; doi:10.1038/s41598-020-71137-0)
Supplement: Supplementary file 1 — Supplementary information [file 41598_2020_71137_MOESM1_ESM.pdf]

A novel immunochromatographic strips assay for rapid and simple detection of systemic lupus erythematosus

Yuhan Sun[1,†], Zhi Li[2,†], Wei Liang[1], Yanlong Zhang[3], Wanli Song[1], Jiazhe Song[1], Kai Xue[1], Meiling Wang[2], Wenying Sun[2], Jianguo Gu[4], Ming Li[1,*], and Wenzhe Li[1,*]

[1]College of Basic Medical Science, Dalian Medical University, 9-Western Section, Lvshun South Road, Dalian, Liaoning 116044, China; [2] Clinical Laboratory, Dalian Municipal Central Hospital, 826-Xinan Road, Shahekou District, Dalian, Liaoning 116033, China; [3] Department of Wildlife Medicine, College of Wildlife Resources, Northeast Forestry University, 26-Hexing Road, Harbin, Heilongjiang 150040,China; [4] Institute of Molecular Biomembrane and Glycobiology, Tohoku Medical and Pharmaceutical University, Sendai, Miyagi 981-8558, Japan

[*] To whom correspondence should be addressed. Tel: +86-411-86118660; Fax: +86-411-86110282; E-mail:vivianmarat@163.com, liwenzhe@dmu.edu.cn

[†] These authors contributed equally to the work.

1. **Supplementary Figure 1：ESI-MS spectrometry of N-glycan structure on the IgG**
2. **Supplementary Figure 2：The full-length blots of Figure 2.**
3. **Supplementary Figure 3：The full-length blots of Figure 3.**
4. **Supplementary Figure 4：The full-length gel and blots of Figure 4.**
5. **Supplementary Figure 5：The full-length gel and blots of Figure 5.**
6. **Supplementary Table 1：Clinical characteristics of patients with autoimmune disease.**
7. **Supplementary Table 2：Clinical characteristics of patients with SLE**

Supplementary Figure 1

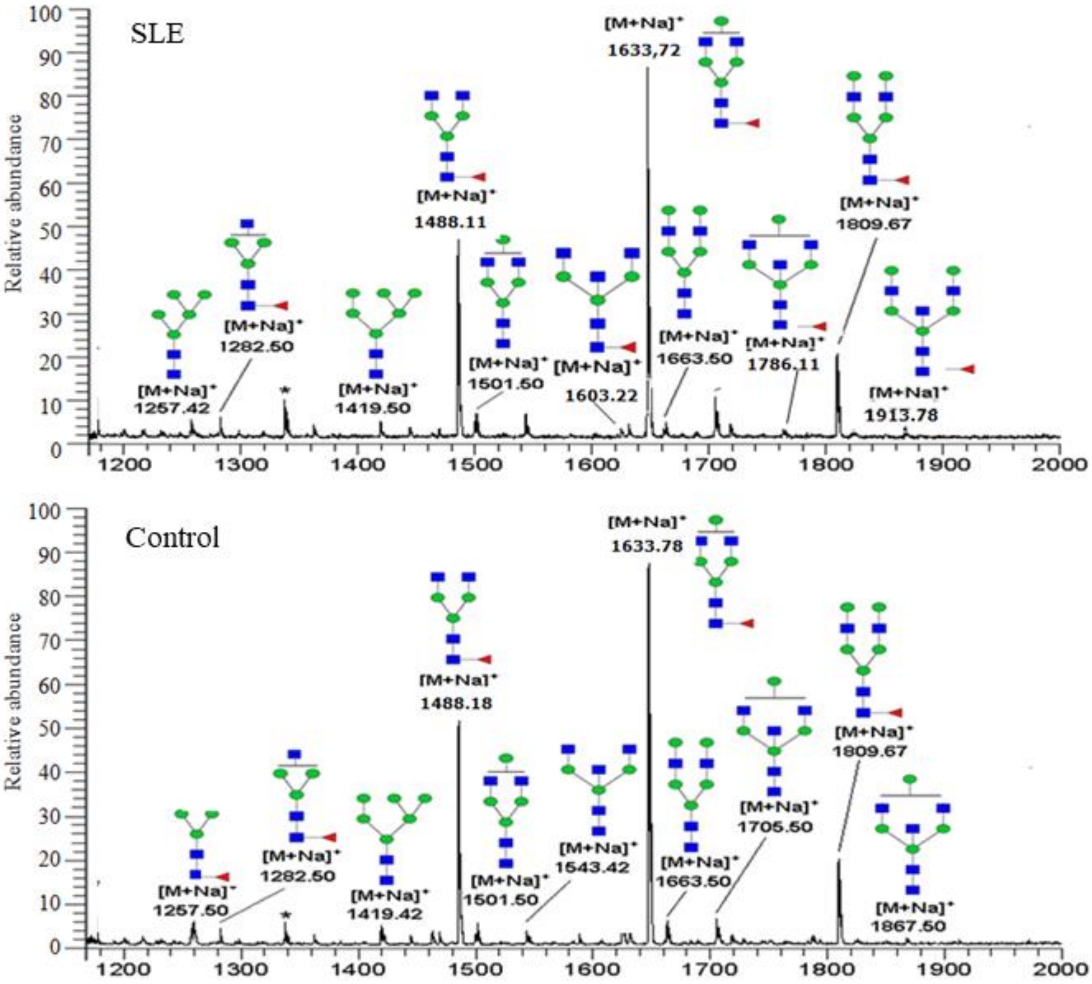

Supplementary Figure 1. ESI-MS spectrometry of N-glycan structure on the IgG in the sera of SLE patients and healthy controls. The serum samples (n =3) were mixed and the N-glycan samples were injected via a Rheodyne loop with a colume of 2 μl and subsequently brought into the electrospray ion source by a stream of 50% methanol (v/v) at a flow rate of 200 μl/min. Triangle, fucose; Square, N-acetylglucosamine; circle, mannose.

Supplementary Figure 2

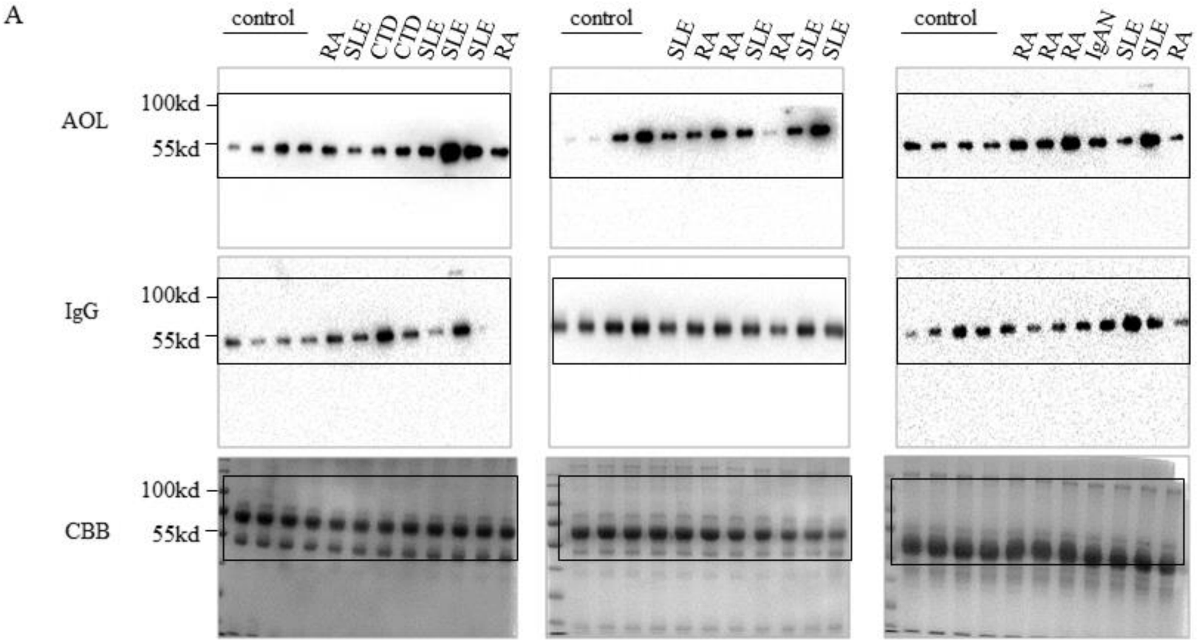

Supplementary Figure 2: The full-length gel and blots of Figure 2. (A) The full-length blots of Figure 2A in this manuscript. Boxed regions were cropped and shown as the figure.

Supplementary Figure 3

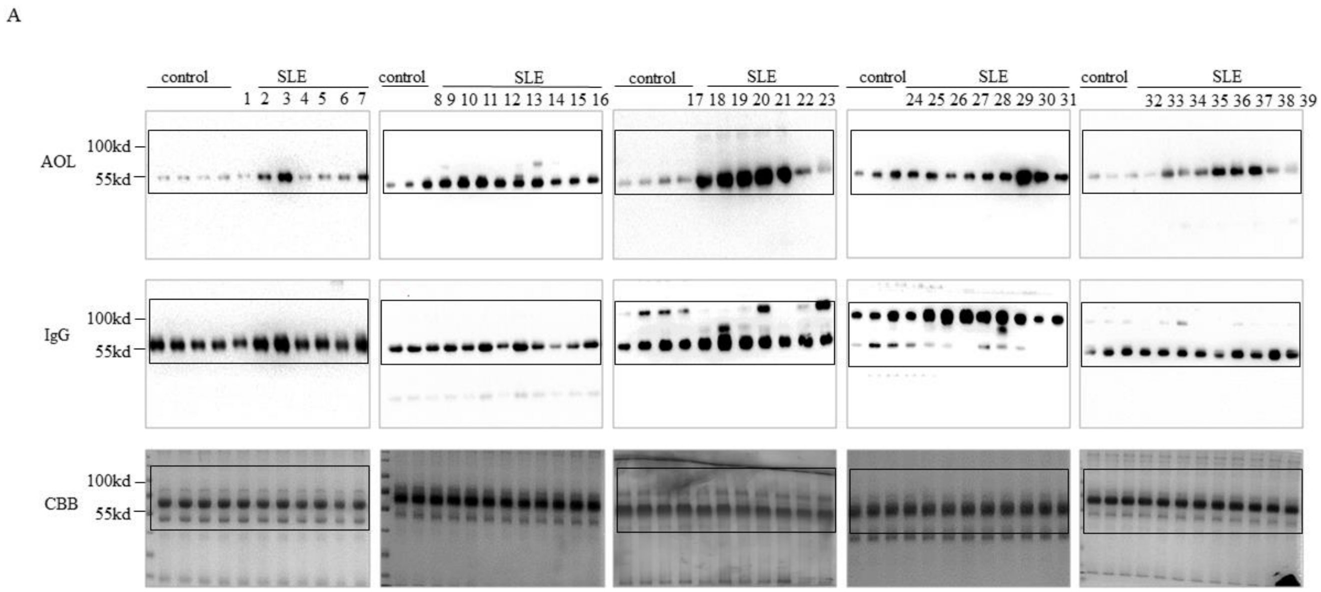

Supplementary Figure 3: The full-length gel and blots of Figure 3. (A) The full-length blots of Figure 3A in this manuscript. Boxed regions were cropped and shown as the figure.

Supplementary Figure 4

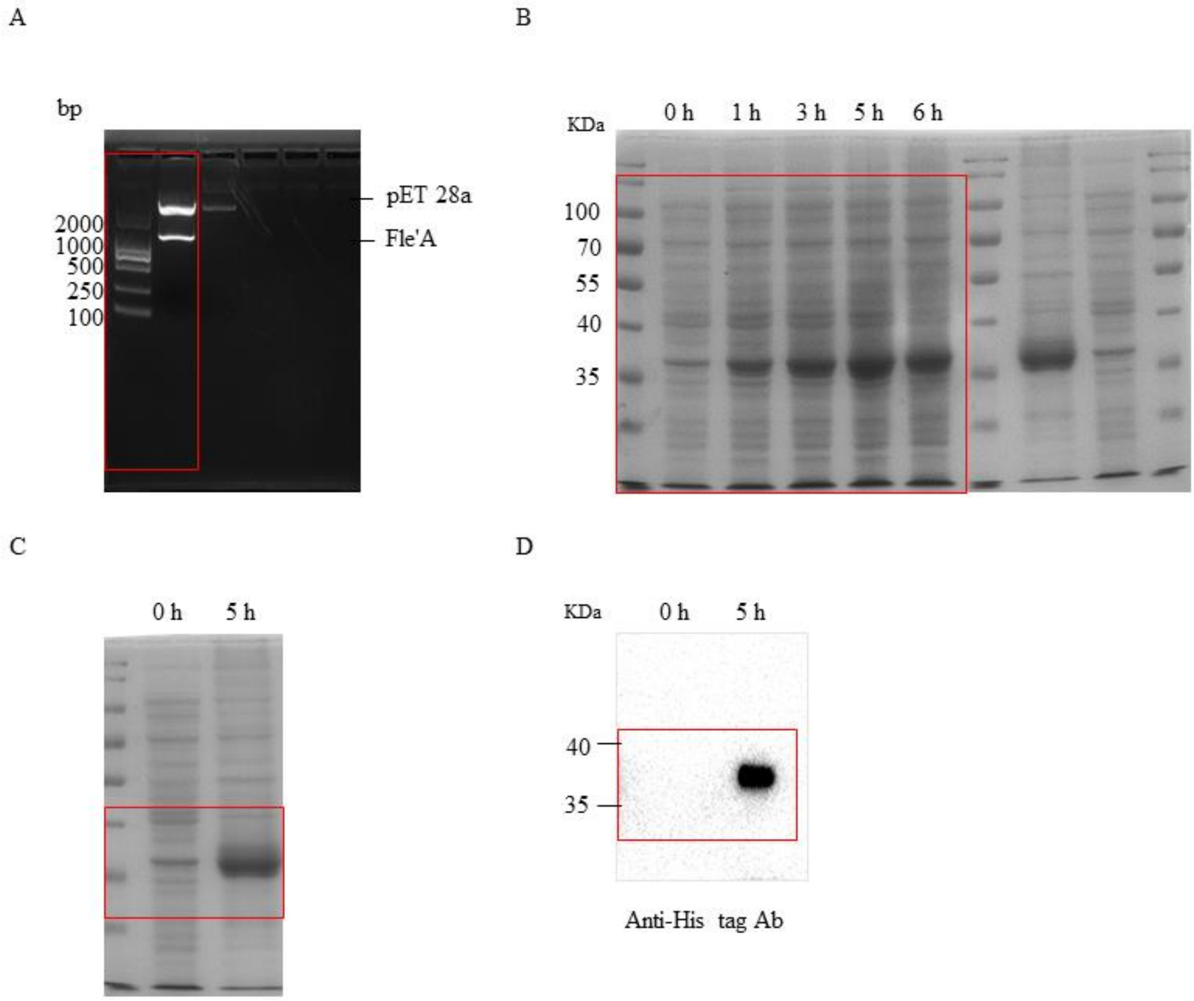

Supplementary Figure 4: The full-length gel and blots of Figure 4. A) a full-length gel of Figure 4 in this manuscript; B-D) a full-length blots of Figure 4C-E in this manuscript. Boxed regions were cropped and shown as the figure.

Supplementary Figure 5

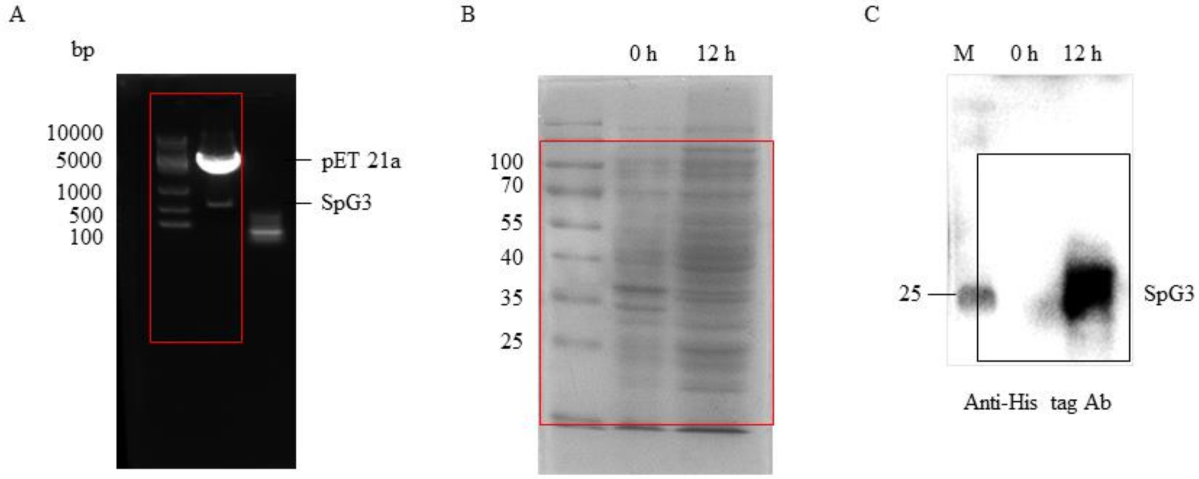

Supplementary Figure 5: The full-length gel and blots of Figure 5. A) a full-length gel of Figure 5B in this manuscript; B,C) a full-length blots of Figure 5C,D in this manuscript. Boxed regions were cropped and shown as the figure.

Supplementary Table 1. Clinical characteristics of patients with autoimmune disease

Patients	Age/Sex/Ethnicity	ANA titer	Disease
1	62/F/Asian	1:100	RA
2	58/F/Asian	1:320	SLE
3	71/F/Asian	——	CTD
4	55/F/Asian	——	CTD
5	68/F/Asian	1:320-1000	SLE
6	35/M/Asian	1:320	SLE
7	65/F/Asian	1:100-320	SLE
8	47/F/Asian	1:100	RA
9	37/F/Asian	1:320	SLE
10	28/F/Asian	1:100	RA
11	66/F/Asian	1:100	RA
12	67/F/Asian	1:1000	SLE
13	28/F/Asian	1:3200	RA
14	61/F/Asian	1:320-1000	SLE
15	71/F/Asian	1:1000	SLE
16	61/M/Asian	1:320	RA
17	67/F/Asian	1:100	RA
18	66/F/Asian	1:320	RA
19	53/F/Asian	1:1000	IgAN
20	29/F/Asian	1:100	SLE
21	37/M/Asian	1:320	SLE
22	59/F/Asian	1:100	RA

Systemic lupus erythematosus, SLE; Rheumatoid arthritis, RA; Connective tissue disease, CTD; IgA nephropathy, IgAN.

Supplementary Table 2. Clinical characteristics of patients with SLE

Patients	Age/Sex/Ethnicity	Fulfilled 1982 ACR criteria	ANA titer	Disease
1	58/F/Asian	3, 7, 9, 10, 11	1:320	SLE
2	68/F/Asian	1, 5, 7, 9, 10, 11	1:320-1000	SLE
3	35/M/Asian	1, 3, 4, 5, 9, 10, 11	1:320	SLE
4	65/F/Asian	7, 9, 10, 11	1:100-320	SLE
5	37/F/Asian	5, 6, 9, 11	1:320	SLE
6	67/M/Asian	3, 5, 6, 9, 10, 11	1:1000	SLE
7	61/F/Asian	1, 3, 4, 10, 11	1:320-1000	SLE
8	71/F/Asian	5, 6, 7, 9, 10, 11	1:1000	SLE
9	61/F/Asian	3, 7, 9, 10, 11	1:320	SLE
10	67/F/Asian	1, 4, 5, 11	1:100	SLE
11	66/F/Asian	1, 4, 7, 9, 10, 11	1:320	SLE
12	29/F/Asian	1, 9, 10, 11	1:100	SLE
13	37/M/Asian	7, 9, 10, 11	1:320	SLE
14	53/F/Asian	1, 3, 5, 7, 9, 10, 11	1:3200	SLE
15	53/F/Asian	1, 5, 6, 7, 9, 10, 11	1:3200	SLE
16	54/F/Asian	3, 4, 10, 11	1:320-1000	SLE
17	59/F/Asian	1, 6, 7, 9, 10, 11	1:1000	SLE
18	68/F/Asian	3, 4, 9, 10, 11	1:100-320	SLE
19	53/F/Asian	1, 7, 9, 10, 11	1:320-1000	SLE
20	68/F/Asian	1, 4, 10, 11	1:100	SLE
21	60/F/Asian	1, 5, 7, 9, 10, 11	1:3200	SLE
22	18/F/Asian	3, 5, 10, 11	1:320	SLE
23	37/M/Asian	3, 5, 6, 9, 10, 11	1:320-1000	SLE
24	21/F/Asian	1, 3, 10, 11	1:100	SLE
25	43/F/Asian	1, 3, 9, 10, 11	1:100	SLE
26	39/F/Asian	1, 5, 9, 10, 11	1:1000	SLE
27	54/F/Asian	5, 7, 9, 10, 11	1:320	SLE
28	20/F/Asian	3, 7, 9, 10, 11	1:320	SLE
29	39/F/Asian	1, 5, 6, 7, 9, 10, 11	1:3200	SLE
30	23/F/Asian	1, 3, 4, 10, 11	1:100-320	SLE
31	59/F/Asian	1, 5, 6, 9, 10, 11	1:1000	SLE
32	46/F/Asian	1, 3, 5, 7, 9, 10, 11	1:3200	SLE
33	36/F/Asian	3, 5, 6, 9, 10, 11	1:1000	SLE
34	52/M/Asian	1, 7, 9, 10, 11	1:1000	SLE
35	52/F/Asian	7, 9, 10, 11	1:1000	SLE
36	52/F/Asian	1, 7, 9, 10, 11	1:320-1000	SLE
37	29/F/Asian	3, 4, 9, 10, 11	1:320	SLE
38	30/F/Asian	5, 6, 9, 11	1:100	SLE
39	50/F/Asian	1, 6, 9, 10, 11	1:320	SLE

1. Lupus malar rash; 2. Classic discoid rash; 3. Photosensitive; 4. Oral ulcers; 5. Synovitis; 6. Serositis; 7. Renal; 8. Neurologic; 9. Hemolytic anemia; 10. Immunologic; 11. ANA level above laboratory reference.
